# First Alaskan records and a significant northern range extension for two species of Diplura (Diplura, Campodeidae)

**DOI:** 10.3897/zookeys.563.6404

**Published:** 2016-02-15

**Authors:** Derek S. Sikes, Robert T. Allen

**Affiliations:** 1 Curator of Insects, University of Alaska Museum, Fairbanks, AK 99775-6960, USA; 2 Adjunct Professor, Mississippi Entomological Museum, Mississippi State University, Starkville MS 39759, USA

**Keywords:** Diplura, Campodeidae, Alaska, new record, soil microarthropods, Protura, Symphyla, Pauropoda, refugium

## Abstract

Species in the class Diplura are recorded from Alaska for the first time. Two species, *Tricampa rileyi* Silvestri from Dall and Prince of Wales Islands in the Alexander Archipelago of Southeast Alaska and *Metriocampa allocerca* Conde & Geeraert from near Quartz Lake, southeast of Fairbanks, both in the family Campodeidae, are documented based on recently collected specimens deposited in the University of Alaska Museum Insect Collection. A brief review of the history of the documentation of the Alaskan soil microarthropod fauna is provided, as well as discussion of possible glacial refugia.

## Introduction

Documentation of the Alaskan entomofauna has accelerated in the twenty-first century, with over 1.2 M specimens cataloged into the UAM Insect Collection since the year 2000 (http://arctos.database.museum/saved/UAM-Insects-since-2000). However, relatively little historic or current attention has been directed at the soil microarthropod fauna of Alaska, which likely contains many new records and species. This is expected, in part, because much of Alaska remained glacier-free during the Tertiary when most of what is now Canada was buried under glaciers (Ives 1974, Matthews 1975, Behan 1978, Pielou 1991). Alaska acted as a refugium for taxa, many of which are potentially endemic (360 arthropod species), some of which were presumably eliminated or prevented from dispersing by glaciers elsewhere. These endemics are often wingless, blind, soil dwelling species such as the beetles *Chionotyphlus alaskensis* (Smetana 1986), *Alaocybites egorovi* Grebennikov (2010) (known in Alaska from a fossil), and *Pinodytes borealis* (Peck & Cook, 2011), new taxa of which continue to be found (e.g. a flightless mecopteran *Caurinus tlagu* Sikes & Stockbridge, 2013). All fifteen species of Protura known from Alaska have yet to be documented occuring outside of Alaska, and are thus potential endemics (Nosek 1977, 1980, Allen 2007). The Pauropod (Myriapoda) fauna of Alaska comprises nine potentially endemic species (Scheller 1986). A single species, among the 11 known for Alaska, of soil centipede (Myriapoda: Geophilomorpha), *Escaryus paucipes* Chamberlin 1946, is a potential Alaskan endemic (Weber 1949). The UAM Insect collection has 10 specimens of Symphyla (Myriapoda) from the northern region of Alaska’s Prince of Wales Island (http://arctos.database.museum/saved/AK-Symphyla), but these remain unidentified and there are no published records of Symphyla from Alaska that we are aware of. Although we have found no published records of Diplura from Alaska there are records of a Dipluran identified as *Tricampa* sp. from Prince of Wales Island recorded in unpublished documents (conference proceedings and US Forest Service reports) prepared by Carlson (1994, 1997, 2005). We have fewer records of potentially endemic Collembola – only two of over 200 species known for Alaska (*Spinonychiurus alaskensis* Pomorksi & Kaprus, 2015 and *Arneria filiformis* Pomorski, 2000). We have started, but not yet finished, summarizing the mite fauna of Alaska based on the works of Behan (1978) and Marshall et al. (1987), in which potential Alaskan endemics are certain to exist. These advances in our understanding of the soil fauna of Alaska demonstrate the progress made since Hilton’s (1931a, b) focused efforts to collect Diplura, Protura, Symphyla, and Pauropoda from Alaska, during which he failed to recover any of these taxa except Pauropoda.

This unique Beringian fauna has received relatively little entomological attention historically (Riegert 1999). No study has yet focused exclusively on these potential endemics. These low-vagility organisms, like those adapted to alpine zones, are of particular concern in a changing climate because dispersal to maintain their ideal conditions is difficult. We know the northernmost latitudes are warming and drying more rapidly than any other region on Earth (Serreze et al. 2000) and alarming ecological and physical changes are being seen in Alaska (Chapin et al. 2006). The boreal forest, which dominates much of this northern landscape, making up about 17% of the earth’s land surface area (Bonan 1992), is the coldest forested biome on Earth and is filled with organisms adapted to low temperatures. Alaska has warmed about 2 °C since the 1950s and 3.5 °C in the interior during the winter (US Global Change Research Program, National Assessment 2001). The growing season has lengthened by about two weeks, shrubs are invading the tundra and alpine zones, fires are more frequent and intense, permafrost and glaciers are melting, and Alaska’s climate is shifting beyond the physiological optimum for one of its dominant boreal forest species, *Picea glauca*, white spruce (Veblen and Alaback 1996, Stone et al. 2002, Lawrence and Slater 2005, Sturm et al. 2005, McGuire et al. 2009, Beck et al. 2011, Juday et al. 2015). Additionally, the increase in extremes of warm temperatures in the boreal forest are associated with rapid maturation and increasingly large outbreaks of wood- and leaf-feeding insects, which increase stress to already moisture-stressed trees. Melting permafrost allows precipitation to drain, thereby drying the soil and further stressing the trees, making them more likely to burn. Post-fire successional trajectories have already begun to shift (Juday et al 2015) – this region will gradually lose its conifers which will be replaced perhaps by something like an aspen parkland – a combination of patches of aspen within a shrub grassland (Hogg and Hurdle 1995). We sit on the edge of this enormous ecological transition unlike anything modern humans have experienced before. It is therefore with great urgency that we document the current entomofauna of Alaska. We herein report on the first records of two species of Diplurans never before documented from Alaska.

## Methods

Specimens of *Tricampa rileyi* were collected primarily by forceps but also one specimen was recovered using Berlese funnels as part of two projects: Effects of forestry practices on ecological indicator species in the Tongass National Forest, Prince of Wales Island, Alaska and Baseline Community Surveys of Alpine and Subalpine Habitats in Southeast Alaska. As part of the Tongass sampling, BioQuip collapsible Berlese funnels were used with ~ 1m^2^ of leaf/moss litter sifted prior to running under 40 watt bulbs for 48h. The Berlese funnel sample came from a low elevation (41-45 m) old growth forest site. The rest of the specimens (n = 40) came from alpine and subalpine habitats between 540 and 881 m elevation. Details of habitat composition and links to photos of each habitat are available in the results below. Specimens of *Metriocampa allocerca* (n = 6) were collected by forceps in a mid-elevation (308 m) spruce forest as part of a general survey of the Quartz Lake entomofauna.

Preparation of specimens for study involved removing specimens from the ethyl alcohol in which they had been stored and mounting them on standard microscope slides. A small drop of mounting medium (polyvinyl alcohol) was placed on the slide, the specimen was then placed in the medium and positioned, and a cover slip added. The slides were allowed to dry for five days on a warm slide dryer. Specimens were then studied at 200× and 400× using a Leica DMKB compound microscope with phase contrast lighting.

All specimens are deposited in the University of Alaska Museum Insect Collection and their data are available online at the links provided below. The data are also shared with iDigBio and GBIF.

## Results

**Figure 1.**
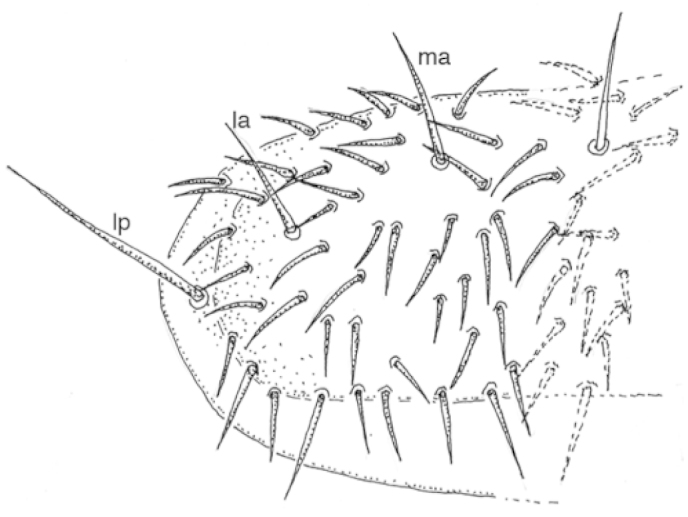
*Tricampa rileyi* Silvestri, pronotum.

**Figure 2.**
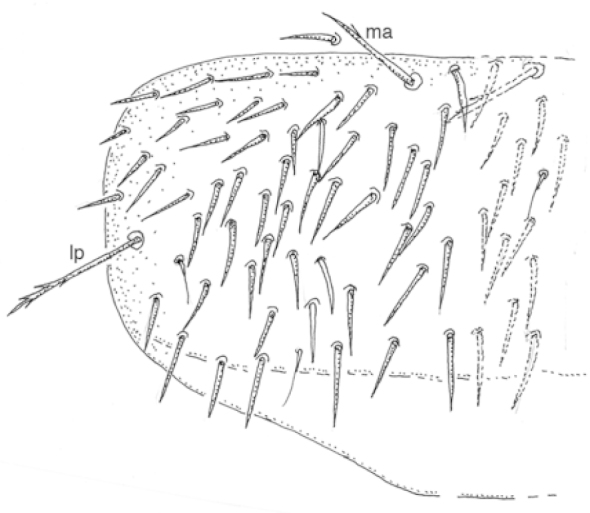
*Metriocampa allocerca* Conde & Geeraert, pronotum.

**Figure 3.**
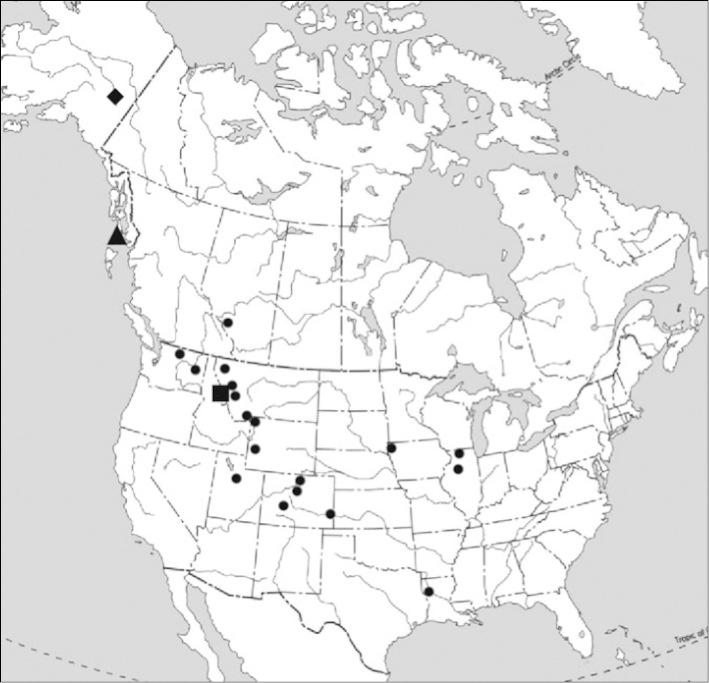
Distribution of *Tricampa rileyi* and *Metriocampa allocerca*: *Tricampa rileyi*, circles = previous distribution, triangle = Alaska; *Metriocampa allocerca*, square = previous distribution, diamond = Alaska distribution.

The two Alaskan species may easily be separated by the number of macrochaeta on the pronotum. Species in the genus *Tricampa* have three macrochaeta (median anterior, *ma*, lateral anterior, *la*, lateral posterior, *lp*) (Fig. 1) while species in the genus *Metriocampa* have only two pronotal macrochaetae (*ma*, *lp*) (Fig. 2). Figure 3 shows the known distribution of the two species. The following list gives most of the specimen data for these species in Alaska – complete data are available at the links provided (Table 1).

**Table 1.** Specimen data.

***Tricampa rileyi* Silvestri**		
Data including habitat photos, available online at: http://dx.doi.org/10.7299/X7JH3M91		
**ALASKA: Dall Island**		
RTA-2014-1	UAM100110978, UAM:Ento:231681	
Dall IsI.	54.99670°N	133.00807°W
subalpine forest, *Abies lasiocarpa*, *Tsuga mertensiana*		
1 male, 4 females		
RTA-2014-5	UAM100110991, UAM:Ento:231694	
Dall IsI.	54.99605°N	133.02089°W
heath, *Empetrum nigrum*, *Philodoce glanduliflora*		
1 specimen SEM		
RTA-2014-6	UAM100110790, UAM:Ento:233667	
Dall IsI	54.99670°N	133.00807°W
floodplain meadow, under rocks		
3 females		
RTA-2014-7	UAM100111001, UAM:Ento:231631	
Dall IsI.	54.99617°N	133.00932°W
flood meadow, *Athyrium*, *Rubus spectabilis*		
1 male 3 females		
RTA-2014-16	UAM100111083, UAM:Ento:232379	
Dall IsI	54.99555°N	133.01039°W
flood meadow, *Athyrium*, *Caltha leptosepala*		
3 males, 5 females		
**ALASKA: Prince of Wales Island**		
RTA-2014-2	UAM100110963, UAM:Ento:217660	
Staney Creek	55.79901°N	133.11782°W
old growth, SEM 1 specimen		
RTA-2014-3	UAM100180086, UAM:Ento:233675	
nr Black Lk	55.58988°N	132.89034°W
rocky heath, *Cassiope mertensiana*, *Luetkea pectinata*, *Harrimanella stelleriana*		
1 male		
RTA-2014-4	UAM100111153, UAM:Ento:232680	
nr Black Lk	55.58988°N	132.89548°W
wet meadow, near bear dung, *Caltha Leptosepala*, *Athyrium filix-femina*		
1 female		
RTA-2014-8	UAM100180094, UAM:Ento:233702	
nr Black Lk	55.590299°N	132.88896°W
meadow, *Nephrophyllidium crista-galli*, *Anemone narcissiflora*		
1 male 2 females		
RTA-2014-17	UAM100111137, UAM:Ento:232618	
nr Black Lk	55.58964°N	132.88783°W
rocky meadow, *Nephrophyllidium crista-galli*, *Luetkea pectinata*		
2 males, 1 female		
RTA-2014-19	UAM100111147, UAM:Ento:232651	
nr Black Lk	55.58898°N	132.88927°W
wet meadow, *Luetkea pectinata*, *Caltha leptosepala*		
4 males, 4 females		
***Metriocampa allocerca* Conde & Geeraert**		
Data, including habitat photo, online at: http://dx.doi.org/10.7299/X7P84C1R		
A video taken by the first author of this species at the Quartz Lake site is available at: https://youtu.be/my25LhHNFbg		
**ALASKA: Quartz Lake**		
RTA-2014-18	UAM100046686, UAM:Ento:241928	
Quartz Lake	64.22086°N	145.80301°W
*Picea*, moss carpet, firepit, under rotting logs, rocks		
3 males, 3 females		

## Discussion

*Tricampa rileyi* Silvestri, 1993 is the most widely distributed among the four North American species in this genus (Allen 1994, 2002). It ranges from Louisiana north to Illinois and Iowa, west into Colorado, Utah, Wyoming, Montana, Washington, and has been recorded from Alberta (Banff), Canada (Allen 2002; Schwaninger 1996). Collections reported herein extend this range into southern Alaska. *Metriocampa allocerca* Conde & Geeraert, 1962 has been recorded from only the type locality in Montana. The new record given here is from just southeast of Fairbanks, Alaska.

Species in both *Metriocampa* and *Tricampa* have been described from the Eastern Hemisphere. Three species of *Metriocampa* are known from Japan and China. One species belonging to the genus *Tricampa* has been recorded from Australia. Diplura found primarily in western North America have not been thoroughly studied nor has the Asian dipluran fauna. It is highly likely that other North America/Asian biogeographic relationships will emerge as the faunae in the two regions become better known. Neither genus is known to occur in Europe but among the diverse European Diplura fauna none of the species have been recorded as far north (64°N) as the locality given for *Metriocampa* here (Paclt 1957). Lagerlöf and Andrén (1991) report on unidentified diplurans from central Sweden (60°N) and Reuter (1895) reported *Campodea staphylinus* Westwood from Kirkkonummi (Kyrkslätt) and Helsinki, Finland (60°N). These new northern records not only add additional distributional and biogeographic data to our knowledge of this group but also add to our knowledge about the environments and habitats Diplura are able to inhabit.

It could be argued that the rarity of diplurans in Alaska may result from a lack of effort spent using appropriate methods of capture. That is, if appropriate effort were expended, they would not be considered rare. We feel this is unlikely, due primarily to the state-wide collecting efforts of the first author, using the same methods which resulted in these two discoveries. Although as yet undocumented dipluran populations may occur in Alaska, given the effort to date, we expect there to be few.

Given that Prince of Wales Island was mostly buried under an ice sheet during the maximum of the late Wisconsin glaciation 26,000 to 13,000 ^14^C years BP (Carrara et al. 2007) and had been repeatedly buried by ice during the Pleistocene, the presence of these low vagility organisms seems unusual. However, there exists considerable biological and geological evidence that suggests ice-free refugia in the Alexander Archipelago may have existed during this time, allowing organisms to survive in relative isolation, and re-seed the region after deglaciation (Carrara et al. 2007). Twenty seven of the 108 mammal species or subspecies occurring in southeastern Alaska are endemic to the area (Cook et al. 2001). *Tricampa rileyi* was recovered from regions that were reconstructed as under ice by Carrara et al. (2007, fig. 3). Post deglaciation dispersal to these sites from ice-free refugia is the most likely explanation.

The *Metriocampa allocerca* specimens were found at a site, Quartz Lake, that has received recent archeological study. Wooller et al. (2012) document the earliest human occupation of these sites at 13,100–12,700 cal yr BP and cite dates for the origin of the gravel terrace which formed the lake to between 140,000 and 40,000 years ago. The disjunct nature of this species with an interior Alaska population and a population in Montana is similar to that of *Thanatophilus coloradensis* (Wickham, 1902) (Coleoptera: Silphidae) – a species known from interior Alaska and northern British Columbia in the north, and from elevations above tree line in Colorado, New Mexico, Utah, Montana and Wyoming in the south, with no intervening records (Anderson and Peck 1985). Genetic data would be needed to determine if this pattern is due to recent dispersal or ancient vicariance.

## References

[B1] Allen RT (1994) An Annotated Checklist and Distribution Records of the subfamily Campodeiinae in North America (Insecta: Diplura: Rhabdura: Campodeidae). Transactions of the American Entomological Society 120(3): 181–208.

[B2] Allen RT (2002) A Synopsis of the Diplura of North America: Keys to Higher Taxa, Systematics, Distributions and Descriptions of New Taxa (Arthropoda: Insecta). Transactions of the American Entomological Society 128(4): 403–466.

[B3] Anderson RS Peck SB (1985) The Carrion Beetles of Canada and Alaska (Coleoptera: Sil- phidae and Agyrtidae). The Insects and Arachnids of Canada, Part 13. Publication 1778, Research Branch Agriculture Canada, Ottawa, 121 pp.

[B4] Allen RT (2007) Studies on the North American Protura 1: catalogue and atlas of the Protura of North America; description of new species; key to the species of Eosentomon. Proceedings of the Academy of Natural Sciences of Philadelphia 156: 97–116.

[B5] Beck PSA Juday GP Alix C Barber VA Winslow SE Sousa EE Heiser P Herriges JD Goetz SJ (2011) Changes in forest productivity across Alaska consistent with biome shift. Ecology Letters 2011: 1–7. doi: 10.1111/j.1461-0248.2011.01598.x 10.1111/j.1461-0248.2011.01598.x 21332901

[B6] Behan VM (1978) Diversity, distribution and feeding habits of North American arctic soil Acari. Unpublished Ph.D. thesis, McGill Univ., Montreal, 428 pp.

[B7] Bonan G (1992) A simulation analysis of environmental factors and ecological processes in North American boreal forests. In: Shugart HH Leemans R Bonan GB (Eds) A systems analysis of the global boreal forest. Cambridge University Press, New York, NY. doi: 10.1017/cbo9780511565489.018

[B8] Carlson KR (1994) Inventory and assessment of ecological relationships between cavernicolous (cave-associated) invertebrate species and their interactions in representative karst ecosystems on carbonate terrain in the Ketchikan area Tongass National Forest. Part I. Dall Island. Karst Biosciences. http://www.researchgate.net/publication/274194363

[B9] Carlson KR (1997) Invertebrate habitat complexity in Southeast Alaskan karst ecosystems. Proceedings of the 1997 karst and cave management symposium and 13th national cave management symposium, 34–43. http://www.researchgate.net/publication/274194352

[B10] Carlson KR (2005) Southeast Alaskan karst-associated invertebrate identifications. Unpublished report http://www.researchgate.net/publication/275584278

[B11] Carrara PE Ager TA Baichtal JF (2007) Possible refugia in the Alexander Archipelago of southeastern Alaska during the late Wisconsin glaciation. Canadian Journal of Earth Science 44: 229–244. doi: 10.1139/E06-081

[B12] Chapin FS III Oswood MW Van Cleve K Viereck LA Verbyla DL (Eds) (2006) Alaska’s Changing Boreal Forest. Oxford University Press, New York, xiv + 354 pp.

[B13] Cook JA Bidlack AL Conroy CJ Demboski JR Fleming MA Runck AM Stone KD MacDonald SO (2001) A phylogeographic perspective on endemism in the Alexander Archipelago of southeast Alaska. Biological Conservation 97: 215–227. doi: 10.1016/S0006-3207(00)00114-2

[B14] Grebennikov VV (2010) First *Alaocybites* weevil (Insecta: Coleoptera: Curculionoidea) from the Eastern Palaearctic: a new microphthalmic species and generic relationships. Arthropod Systematics Phylogeny 68(3): 331–365

[B15] Hilton WA (1931a) Pauropoda in Alaska. Science 74: 338. doi: 10.1126/science.74.1918.338 10.1126/science.74.1918.338 17732302

[B16] Hilton WA (1931b) Pauropoda from Alaska and the Yukon. Can. Ent. 63: 280–284. doi: 10.4039/Ent63280-12

[B17] Hogg EH Hurdle PA (1995) The aspen parkland in western Canada: a dry-climate analogue for the future boreal forest? Water, Air and Soil Pollution 82: 391–400.

[B18] Ives JD (1974) Biological refugia and the nunatuk hypothesis. In: Ives JD Barry RG (Eds) Arctic and Alpine Environments. Metheun, London, 605–636.

[B19] Juday GP Alix C Grant TA III (2015) Spatial coherence and change of opposite white spruce temperature sensitivities on floodplains in Alaska confirms early-stage boreal biome shift. Forest Ecology and Management 350(2015): 46–61. doi: 10.1016/j.foreco.2015.04.016

[B20] Lagerlöf J Andrén O (1991) Abundance and activity of Collembola, Protura and Diplura (Insecta, Apterygota) in four cropping systems. Pedobiologia 35: 337–350.

[B21] Lawrence DM Slater AG (2005) A projection of severe near-surface permafrost degradation during the 21st century. Geophysical Research Letters 32: . doi: 10.1029/2005GL025080

[B22] Marshall VG Reeves RM Norton RA (1987) Catalogue of the Oribatida (Acari) of Continental United States and Canada. Memoirs of the Entomological Society of Canada 139: 1–623. doi: 10.4039/entm119139fv

[B23] Matthews JV Jr. (1975) Insects and plant macrofossils from two quaternary exposures in the old crow-porcupine region, Yukon Territory, Canada. Arctic and Alpine Research 7: 249–259. doi: 10.2307/1550000

[B24] McGuire AD Anderson LG Christensen TR Dallimore S Guo L Hayes DJ Heimann M Lorenson TD Macdonald RW Roulet N (2009) Sensitivity of the carbon cycle in the Arctic to climate change. Ecological Monographs 79(4): 523–555. doi: 10.1890/08-2025.1

[B25] Nosek J (1977) A new genus and six new species of Protura from Alaska (Protura: Acerentomidae, Eosentomidae). Entomologica Scandinavica 8: 271–284. doi: 10.1163/187631277X00378

[B26] Nosek J (1980) A new genus and five species of Protura from Alaska. Insect Systematics & Evolution 11(3): 265–273. doi: 10.1163/187631280794824712

[B27] Paclt J (1957) Diplura. Genera Insectorum de P. Wytsman. Fascicule 212E, 123 pp.

[B28] Peck SB Cook J (2011) Systematics, distributions and bionomics of the Catopocerini (eyeless soil fungivore beetles) of North America (Coleoptera: Leiodidae: Catopocerinae). Zootaxa 3077: 1–118

[B29] Pielou EC (1991) After the Ice Age: The return of life to glaciated North America. The University of Chicago Press, 366 pp. doi: 10.7208/chicago/9780226668093.001.0001

[B30] Pomorski R (2000) *Arneria*: A new genus of North American Onychiuridae (Collembola), with a description of two new species. Entomologica Scandinavica 31(3): 317–322. doi: 10.1163/187631200X00066

[B31] Reuter OM (1895) Apterygogenea Fennica. Finlands Collembola och Thysanura. Acta Societatis Pro Fauna et Flora Fennica, XI, 4: 1–35.

[B32] Riegert PW (1999) The Survey of Insects of Northern Canada 1947–1962. Rempeck Publ., SK, Entomological Series No. 8, 49 pp.

[B33] Romuald J Pomorski R Kapruś IJ (2015) Revision of the genus *Spinonychiurus* Weiner 1996 (Collembola: Onychiuridae) with description of five new species. Zootaxa 3914(2): 101. doi: 10.11646/zootaxa.3914.2.1 10.11646/zootaxa.3914.2.1 25661933

[B34] Scheller U (1986) Beringian Pauropoda (Myriapoda). Entomologica Scandinavica 17: 363–391. doi: 10.1163/187631286X00297

[B35] Schwainger D (1996) A Revision of the North American Species in the Genus *Metriocampa* (Campodeidae: Diplura). A thesis submitted in partial fulfillment of the requirements for the degree of Master of Science, University of Delaware, 1996, 99 pp.

[B36] Serreze MC Walsh JE Chapin FS III Osterkamp T Dyurgerov M Romanovsky V Oechel WC Morison J Zhang T Barry RG (2000) Observational evidence of recent change in the northern high-latitude environment. Climatic Change 46: 159–207. doi: 10.1023/A:1005504031923

[B37] Sikes DS Stockbridge J (2013) Description of *Caurinus tlagu*, new species, from Prince of Wales Island, Alaska (Mecoptera, Boreidae, Caurininae). ZooKeys 316: 35–53. doi: 10.3897/zookeys.316.5400 23878513 10.3897/zookeys.316.5400 PMC3713333

[B38] Smetana A (1986) *Chionotyphlus alaskensis* n. g., n. sp., a Tertiary relict from unglaciated interior Alaska (Coleoptera, Staphylinidae). Nouvelle Revue d’Entomologie (N. Sér.) 3(2): 171–187.

[B39] Stone RS Dutton EG Harris JM Longenecker D (2002) Earlier spring snowmelt in northern Alaska as an indicator of climate change. Journal of Geophysical Research 107: . doi: 10.1029/2000JD000286

[B40] Sturm M Schimel J Mechaelson G Welker JM Oberbauer SF Liston LE Fahnestock J Romanovsky VE (2005) Winter biological processes could help convert Arctic tundra to shrubland. BioScience 55: 17–26. doi: 10.1641/0006-3568(2005)055[0017:WBPCHC]2.0.CO;2

[B41] US Global Change Research Program, National Assessment (2001) Overview: Alaska. http://www.globalchange.gov/component/content/article/52-reports-and-assessments/476-overview-alaska

[B42] Veblen TT Alaback PB (1996) A comparative review of forest dynamics and disturbance in the temperate rainforests of North and South America. In: Lawford RG Alaback PB Fuentes E (Eds) High-latitude rainforests and associated ecosystems of the west coast of the Americas: climate, hydrology, ecology, and conservation. Springer, New York, 173–213. doi: 10.1007/978-1-4612-3970-3_9

[B43] Weber NA (1949) Late summer invertebrates, mostly insects, of the Alaskan Arctic Slope. Entomological News 60: 118–128.

[B44] Wooller MJ Kurek J Gaglioti BV Cwynar LC Bigelow N Reuther JD Gelvin Reymiller C Smol JP (2012) An ~ 11,200 year paleolimnological perspective for emerging archaeological findings at Quartz Lake, Alaska. Journal of Paleolimnology 48(1): 83–99. doi: 10.1007/s10933-012-9610-9

